# Comparison of Plaque Accumulation Between Titanium and PEEK Healing Abutments

**DOI:** 10.3390/jfb15110334

**Published:** 2024-11-07

**Authors:** Suphachai Suphangul, Patr Pujarern, Dinesh Rokaya, Chatruethai Kanchanasobhana, Pimduen Rungsiyakull, Pisaisit Chaijareenont

**Affiliations:** 1 Department of Prosthodontics, Faculty of Dentistry, Chiang Mai University, Chiang Mai 50200, Thailand; suphachai_sup@cmu.ac.th (S.S.); chatruethai.kan@cmu.ac.th (C.K.); pimduen.rungsiyakull@cmu.ac.th (P.R.); 2 Department of Advanced General Dentistry, Faculty of Dentistry, Mahidol University, Bangkok 10400, Thailand; patr.puj@mahidol.ac.th; 3 Clinical Sciences Department, College of Dentistry, Ajman University, Ajman P.O. Box 346, United Arab Emirates; d.rokaya@ajman.ac.ae; 4 Center of Medical and Bio-Allied Health Sciences Research, Ajman University, Ajman P.O. Box 346, United Arab Emirates

**Keywords:** dental implant, implant prostheses, healing abutment, poly-ether-ether-ketone, polyetheretherketone, PEEK, titanium, biofilm, plaque accumulation

## Abstract

Titanium (Ti) is considered the gold standard material for provisional implant restorations. Polyetheretherketone (PEEK), a polymeric thermoplastic material, has been progressively used in prosthetic, restorative, and implant dentistry. Recently, PEEK has been used in implant dentistry as a provisional implant restoration. Plaque accumulation and biofilm formation become the major concerns when infection and inflammation occur in the peri-implant tissue. Few reports were studied regarding the biofilm formation on the PEEK surface. This study aimed to compare plaque accumulation between the PEEK and Ti healing abutments. In an in vitro setting, the Ti healing abutment and PEEK healing abutment were subjected to biofilm formation; the result was collected after 24 h, 48 h, 72 h, and 7 days. Biofilms were studied following staining with crystal violet. The data were analyzed by Two-Way ANOVA. It was found that between Ti healing abutment and PEEK healing abutment materials, the biofilm formation on the PEEK surface is slightly higher than Ti, but no statistical difference (*p* > 0.05) was found. The results suggested that plaque accumulation between the Ti healing abutment and the PEEK healing abutment was not different. We concluded that the plaque accumulation on the surface PEEK healing abutment was similar to the conventional Ti healing abutment materials. Hence, both the PEEK and Ti healing abutments can be used as a healing abutment biomaterial according to the requirements of the prostheses in implant dentistry.

## 1. Introduction

Dental implants are a popular treatment for missing teeth either using removable or fixed prostheses. Following the implant placement, a healing abutment is used to cover the fixture. The healing abutment helps in the healing of the soft and hard tissues around the implant surface. Once the healing is completed, osseointegration will be accomplished in three months, before placing the final implant abutment with a prosthetic restoration. A gold standard material of healing abutments these days is Titanium (Ti), due to its great corrosion resistance property in a physiological environment, which results in a passive, stable oxide coating. The gold standard material for healing abutment is Ti. A recent study showed that the customized Ti healing abutment helps to achieve the optimal peri-implant mucosa and supports soft tissue management [1].

**Figure 1:**
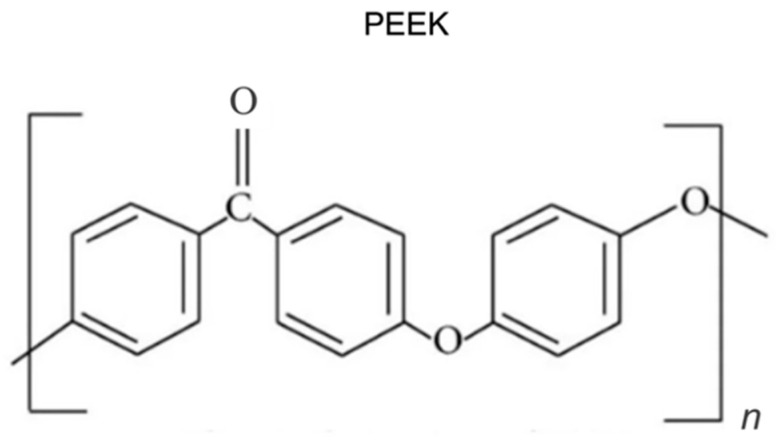
Structure of PEEK.

Polyetheretherketone (PEEK), a polymeric biomaterial, is one of the family members of the polyaryletherketone (PAEK) family (Figure 1) [2,3,4]. PEEK presents ultra-high performance with excellent physical and chemical properties [5,6]. Furthermore, PEEK shows excellent biocompatibility, physical, and mechanical properties for biomedical applications [7,8,9]. These outstanding outcomes of PEEK have attracted it in medicine and dentistry [10,11,12,13,14,15].

Recently, PEEK has been increasingly used as a provisional implant restoration in implant dentistry [16,17]. PEEK healing abutment can be reshaped to be adaptable to different root morphology, which can create a better emergence profile [18,19]. PEEK healing abutments have various advantages and can be used using digital impressions [20]. Moreover, digital impressions using direct intraoral scanning are available in the PEEK healing abutment [11]. This method can reduce the biological trauma of peri-implant tissue due to the continual removal of temporary abutments for conventional impressions [21].

Plaque accumulation and biofilm formation become the major concerns when infection and inflammation occur in the peri-implant tissue. Various factors that can affect the biofilm formation on abutments include surface roughness, wettability, surface energy, and the intrinsic properties of the materials [22,23,24,25,26]. Furthermore, the bacterial adhesion and the biofilm formation on biomaterial depends on hydrophobicity and surface smoothness [25]. It has been also found that the surface chemistry and the implant-abutment design features’ configuration play an important role in biofilm formation [27]. The microgap size between the various components, the surface roughness of the restorations and abutments, the plasma-sprayed coatings exposure and threaded implant surfaces, and the over-contouring of implant restorations lead to plaque accumulation and bacterial colonization [28]. Nevertheless, there are only a few studies regarding the ability of PEEK healing abutments to resist biofilm formation. The null hypothesis was that Ti presents similar bacterial adhesion compared to the PEEK abutment. Thus, this novel study aimed to compare plaque accumulation between Ti and PEEK healing abutments after incubation in an anaerobic chamber for 24 h, 48 h, 72 h, and 7 days at various magnifications.

## 2. Materials and Methods

### 2.1. Healing Abutments

**Figure 2:**
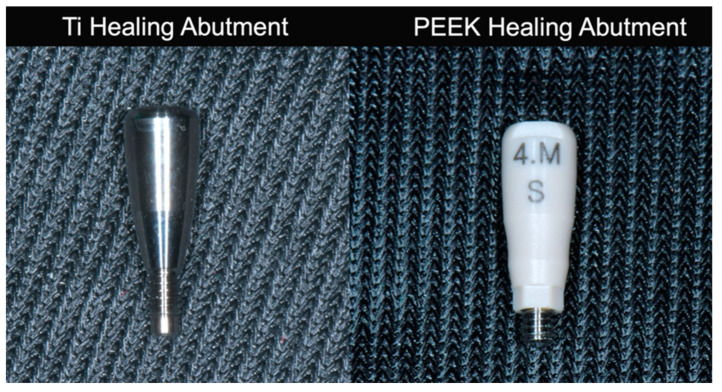
Titanium healing and PEEK healing abutments used in this study.

Twenty-eight Ti healing abutments (Dentium Co., Seoul, Republic of Korea) of diameter 4.0 mm and height 5 mm) and twenty-eight PEEK healing abutments (Dentium Co., Seoul, Korea) of diameter 4 mm and height 6 mm (hex type) were used in this study (Figure 2).

We used an intraoral scanner (Medit i 500, Seoul, Republic of Korea) to scan the 5 random healing abutments connected to each implant analog group (Ti and PEEK) and calculated surface area by Ansys SpaceClaim 3D modeling software. We use the mean of 5 abutment’s surface (mm^2^) to represent each group.

### 2.2. Subgingival Bacterial Collection and Processing

Following the permission from the Ethical Committee of the Faculty of Dentistry/Faculty of Pharmacy, Mahidol University, subgingival plaque collection was performed using a periodontal curette at the distal site of a lower molar from a healthy volunteer (researcher SS) with normal oral hygiene and no underlying medical conditions.

The collected sample was then inoculated to an anaerobe basal broth and incubated in an anaerobic jar with AnaeroPack^TM^ for 24 h. After 24 h, an anaerobe basal broth was adjusted to 0.5 McFarland concentration by using a densitometer device (DEN-18 McFarland Densitometer, Biosan, Riga, Latvia). This variant of turbidity measurements is the McFarland standard [29,30]. The inoculation to an anaerobe basal broth and 0.5 McFarland standard gave the most consistent counts for the bacterial counts and the highest numbers as in other studies [31,32].

### 2.3. Saliva Collection and Processing

A 10 mL saliva sample was taken from the same volunteer (researcher SS). Then, the saliva sample was centrifuged by Centrifuge 5804 R (Eppendorf, Hamburg, Germany) at 5000 rpm for 15 min and only the supernatant was transferred to another container. The supernatant was then diluted with distilled water to 50% concentration, and 1 mL of the supernatant was pipetted into a test tube containing the healing abutment sample. The healing abutments were left in the saliva solution for 24 h at 37 °C.

### 2.4. Biofilm Formation of Subgingival Plaque on Healing Abutments

Ti and PEEK healing abutments were placed in a 24-well plate. Then, 1000 μL of the 0.5 McFarland anaerobe basal broth was added to each well containing the healing abutment. The 24-well plates were then placed in an anaerobic jar with AnaeroPack^TM^ to incubate in an anaerobic chamber for 24, 48, 72 h, and 7 days (n = 7/group). The control wells are subgingival plaque without healing abutments. After incubation, the healing abutments were removed from the well and rinsed once with phosphate-buffered saline (PBS) to remove the loosely attached bacteria. The healing abutments were removed from the media and the biofilms on the abutments were observed under a Stereomicroscope (Leica Microsystems, Wetzler, Germany) at a magnification of ×13.

In addition, scanning electron microscopy (SEM) images of the Ti healing abutments and PEEK healing abutments were taken after incubation in an anaerobic chamber for 24 h, 48 h, 72 h, and 7 days. At first, the healing abutments were dried at room temperature for 24 h, the specimens were mounted on aluminum stubs, and the specimen surfaces were rendered to conduct by applying thin layers of vacuum evaporation of 100–300 Å gold with an ion sputter coater, and viewed with a JEOL JSM- 6610 LV SEM [33] at three magnifications (×18, ×150, and ×5000).

### 2.5. Assessment of the Biofilm on Healing Abutments

The quantity of the biofilms on the abutments was measured by crystal violet staining (PhytoTechnology Laboratories, Overland Park, KS, USA). The crystal violet stain (1%) was used to stain the abutments for 15 min. After that, all healing abutments were moved to a 24-well plate to be rinsed with 600 μL PBS one time and left to dry in the same cabinet. Once dry, some random samples from each group were investigated under a stereomicroscope. The healing abutments were then placed into each glass test tube together with a round glass bead and 600 μL of 20% acetone in ethanol solution was added. The glass tubes were put on a vortex mixer for 5 min. Finally, three 100 μL of each sample were pipetted into a 96-well plate and the optical density at the 570 nm wavelength was measured using a microplate reader (Synergy LX, Biotek, Winooski, VT, USA). The OD of each sample was calculated by averaging three readings of the same specimen and subtracting by the OD of the negative control group. We used new healing abutments in both groups without plaque and performed the same OD procedure to use as a base control. The negative control was zero as there was no crystal violet in these groups.

### 2.6. Statistical Analysis

All data were analyzed using the SPSS 18 Statistics (IBM Corp., Chicago, IL, USA). Descriptive statistics were calculated. The data were compared among two groups (Ti vs. PEEK healing abutment) and between each time (24 h, 48 h, 72 h, and 7 days) using Two-Way ANOVA. The significance level considered in the experiments was α = 0.05.

## 3. Results

**Figure 3:**
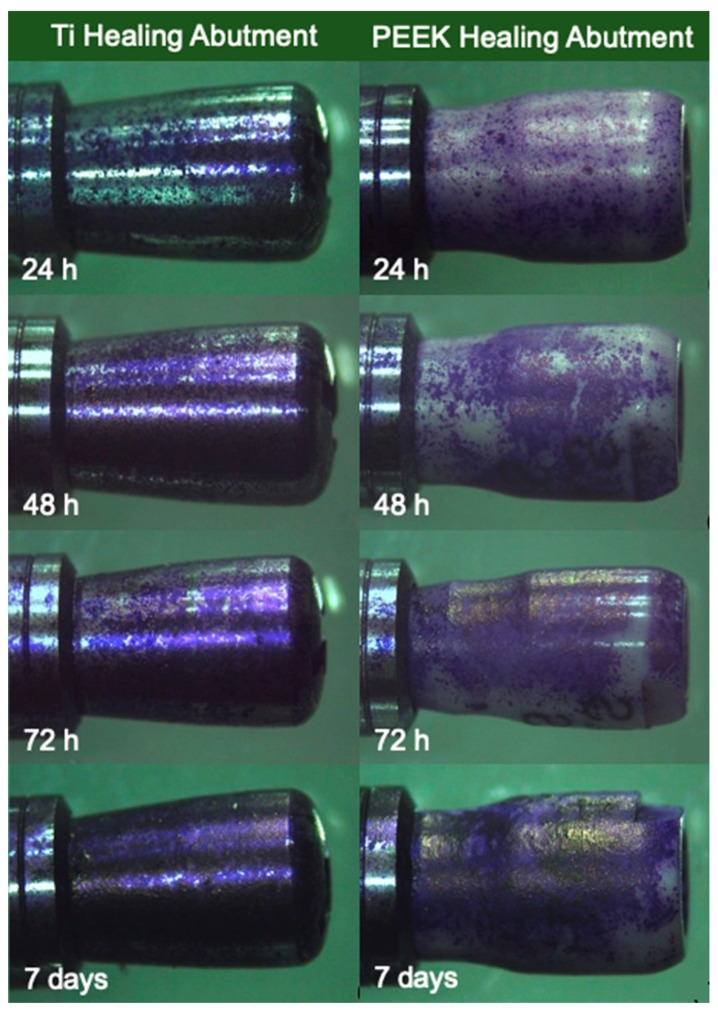
Biofilm on Ti healing abutment and PEEK healing abutment surface dyed with crystal violet at 24 h, 48 h, 72 h, and 7 days.

The surface area of the Ti healing abutment was 81.98 mm^2^, whereas the surface area of the PEEK healing abutment was 93.35 mm^2^. Figure 3 shows the biofilm formation on the Ti healing abutment and PEEK healing abutment following the incubation in an anaerobic chamber for 24 h, 48 h, 72 h, and 7 days.

**Figure 4:**
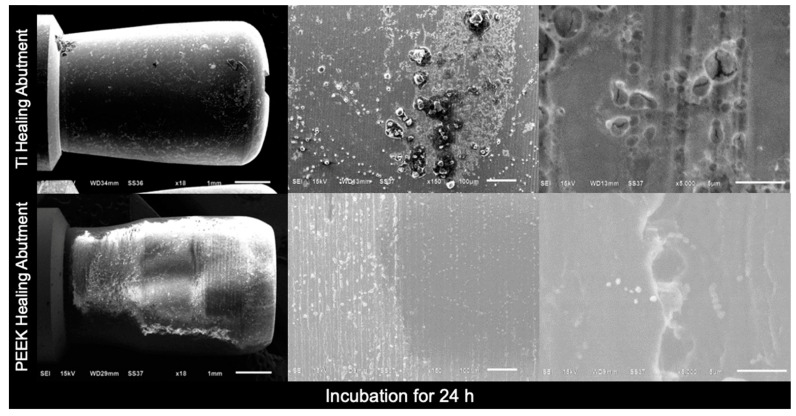
Scanning electron microscopy images of the Ti healing abutment and PEEK healing abutment after incubation in an anaerobic chamber for 24 h at various magnifications (×18, ×150, and ×5000).

**Figure 5:**
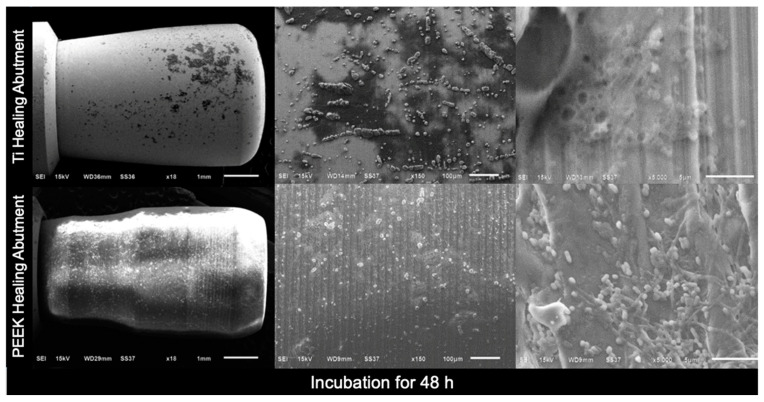
Scanning electron microscopy images of the Ti healing abutment and PEEK healing abutment after incubation in an anaerobic chamber for 48 h at various magnifications (×18, ×150, and ×5000).

**Figure 6:**
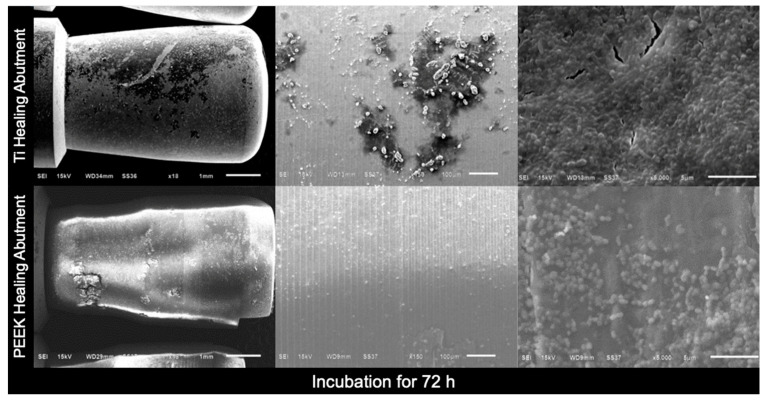
Scanning electron microscopy images of the Ti healing abutment and PEEK healing abutment after incubation in an anaerobic chamber for 72 h at various magnifications (×18, ×150, and ×5000).

**Figure 7:**
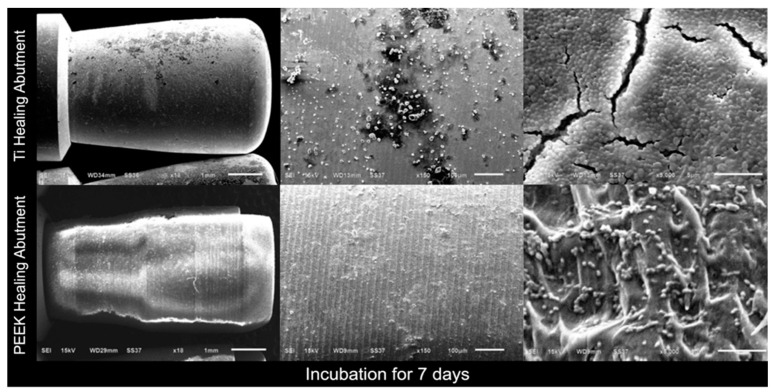
Scanning electron microscopy images of the Ti healing abutment and PEEK healing abutment after incubation in an anaerobic chamber for 7 days at various magnifications (×18, ×150, and ×5000).

Figure 4, Figure 5, Figure 6 and Figure 7 show the SEM images of the Ti healing abutment and PEEK healing abutment after being incubated in an anaerobic chamber for 24 h, 48 h, 72 h, and 7 days at various magnifications (×18, ×150, and ×5000). After each healing abutment was dyed with crystal violet, a stereomicroscope (at a magnification of ×13) was used to observe the biofilm shown in Figure 3. At 24 h, both Ti and PEEK demonstrated a thin layer of biofilm. Then, abundant biofilms were found after 7 days, when compared to 24, 48, and 72 h. The results showed that biofilms on the surface increased over time and the same with biofilms shown on the surface of healing abutments in SEM (Figure 4, Figure 5, Figure 6 and Figure 7).

The amount of biofilms on the healing abutments was expressed as the values of opacity density (OD) at 570 nm per surface area, as shown in Table 1.

**Figure 8:**
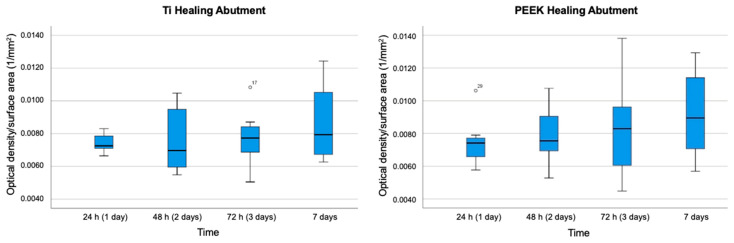
Box-plot graph of biofilm accumulation on Ti healing abutments and on PEEK healing abutments expressed as optical density (OD) values at 570 nm per surface area (1/mm^2^, n = 7).

**Figure 9:**
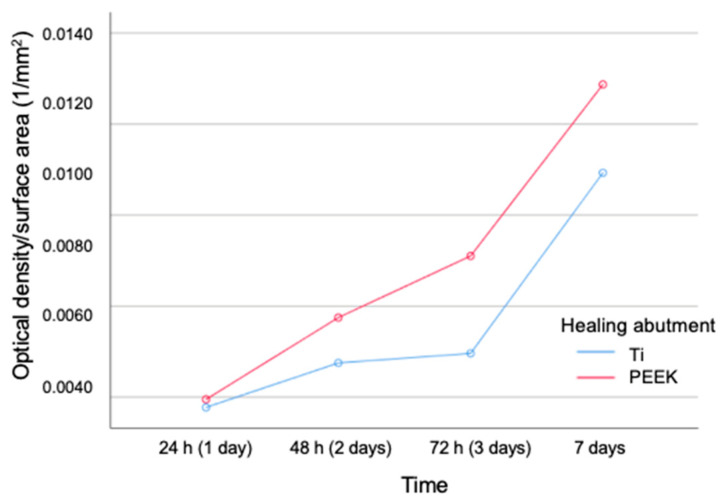
Linear graph of biofilm formation expressed as optical density (OD) per surface area as opposed to time.

Figure 8 shows the box plots showing the results of biofilm accumulation on Ti healing abutments and on PEEK healing abutments expressed as OD values at 570 nm per surface area. Figure 9 shows a linear graph of biofilm formation expressed as OD per surface area as opposed to time.

The results of the Two-Way ANOVA with post hoc analysis are shown in Table 2. They showed that there was not statistical significance in abutment, day, and abutment * day.

## 4. Discussion

Healing abutments are vital components in the healing process that promote the immune responses of bone and mucosa around the implant and improve aesthetic outcomes [34,35,36]. Recently, PEEK healing abutments have been widely used due to their physical properties for biomedical applications [10,16,37]. This study compared the plaque accumulation between conventionally Ti healing abutments and PEEK healing abutments. The null hypothesis was rejected as the plaque accumulation between the Ti healing abutments and PEEK healing abutments.

The results of plaque accumulation on PEEK in our studies were similar to the study conducted by Hahnel et al. [38] and Fernández-Osorio et al. [22]. Hahnel et al. [38] found that the biofilm formation on the PEEK surface was equal to or less than on the surface of conventional Ti and zirconia abutments. They found that there was a significant difference in cell viability which signifies differences in multispecies biofilm formation in different materials after 20 h. However, after 44 h, the cell viability showed no differences between the studied materials. However, this study used disc-shaped specimens. Similarly, Fernández-Osorio et al. [22] studied the effect of surface characteristics and bacterial biofilm developed on the PEEK and Ti healing abutments and they found that the bacterial viability of the biofilm did not show a direct relation to the PEEK and Ti healing abutments.

As a polymer, PEEK used more complex molding techniques than Ti to create a healing abutment. As described earlier, the difference in surface area may affect our results. Our study solved this problem by using intraoral scanning to calculate surface area, so the result was opacity density (OD) per surface area (mm^2^).

Barkarmo et al. [23] found that the formation of biofilm of different reference strains on Ti and PEEK surfaces had no significant differences. At 72 h, *S. sanguinis* initially showed more biofilm formation on PEEK than Ti, but after 120 h, there were no significant differences between the groups, whereas *S. oralis* showed no significant difference in the formation of biofilm on Ti and PEEK surfaces after 72 h and 120 h. With *E*. *faecalis*, there was significantly more biofilm on PEEK compared with Ti after 72 h. Hence, they studied single species separately and only a few species had different results [23]. However, our study involves more complex, multispecies biofilms in the presence of saliva to simulate the oral cavity environment. Covani et al. [39] studied the distribution of bacteria on surfaces of failed implants using histologic analysis. They found that the level of abutment/implant interface presented heavy bacterial colonization, especially in two-stage implants. These findings show that the bacteria can penetrate the micro-gap and cause bacterial colonization and bone loss.

Milinkovic et al. [37] analyzed and compared the soft tissue response to Ti and PEEK healing abutments from histological and immunohistochemical analyses and they found that both PEEK and Ti healing abutments triggered inflammation but the PEEK healing abutments triggered a more intense tissue inflammatory response mediated by plasmacytes and histocytes and activation, whereas the Ti healing abutments triggered the inflammatory response of lower intensity mediated by B-cells. However, clinically, the soft tissues around both Ti and PEEK healing caps were free of inflammation. Hence, the histologic results may be applicable clinically and always need clinical evaluation rather than only histologic evaluation.

Mangano et al. [40] conducted a study on 15 edentulous patients and they were rehabilitated with an implants-supported maxillary overdenture using CAD/CAM fabricated PEEK except there were no implants lost and an 80% success rate [40]. The advantages of PEEK include high wear resistance but low abrasion to the enamel. Similarly, Wimmer et al. [41] found substantially higher wear resistance for PEEK compared to the poly (methyl methacrylate) and nanohybrid composite materials when loaded with enamel antagonists.

Oral biofilm is formed by multi-species and a complex process. Within the limitations of this study, the results cannot completely simulate the clinical setting. Nevertheless, several factors that affected biofilm formation have been created, including acquired pellicle on the surface of the healing abutment and the use of multi-species subgingival plaque from one donor, which decreased the confounding factor, to simulate the same environment as the oral cavity in one patient. Brum et al. [42] mentioned that pure PEEK is susceptible to biofilm formation and presented several strategies to improve antimicrobial and antibiofilm properties, which include the coatings, incorporation of fillers or reinforcement agents to produce nanocomposites and/or blends, incorporation of therapeutic and/or bioactive agents in the matrix or on the surface, and the sulfonation process. In addition, biofilm formation is not the only factor to consider upon choosing a material for healing abutment in treatment. The shape of the healing abutment has a significant role in peri-implant tissue health. Some studies reported that the anatomic healing abutment reduces bone resorption compared to the concave-straight abutment [20]. The shape of the healing abutment is also an important factor that influences tissue healing and aesthetic outcomes [43,44]. Some previous studies stated that using a conventional healing abutment results in a larger size of peri-implant soft tissue contour [18,45,46]. This causes difficulties in subsequent impression-making and insertion of provisional restoration. 

In a study by Beretta et al. [18], comparing peri-implant tissue conditions between standard and modified shapes of PEEK healing abutment, using CAD/CAM technology, the customized group showed less pain score and required fewer steps for prosthetic finalization, creating a better emergence profile. A case report by Pow and McMillan [19] said that the soft tissue contour made by conventional healing abutment resulted in difficulty in the insertion of an anatomically shaped abutment.

In this study, we did not conduct the identification of the different microbiomes due to time limitations. Hence, further tests are needed to identify the accumulation of different microbiomes on different materials, for example, by using the checkerboard DNA–DNA hybridization method. In addition, further studies can be conducted in the clinical setting in more clinical samples considering more confounding factors. In addition, it would be worth investigating what types of microorganisms colonize the abutments because this will also affect the number of colonies and growth conditions. 

## 5. Conclusions

Within the limitations of this study, both the PEEK healing abutment and Ti healing abutment showed that biofilms increased over time on the surface of healing abutments and there was no difference in the plaque accumulation on the surface of the PEEK healing abutment compared to the Ti healing abutment materials. Hence, both the PEEK and Ti healing abutments can be used as a healing abutment biomaterial according to the requirements of the prostheses in implant dentistry.

## Figures and Tables

**Table 1:** Opacity density (OD) per surface area (mm^2^) of Ti and PEEK healing abutment of 4 different groups: 24 h, 48 h, 72 h, and 7 days.

	24 h		48 h		72 h		7 days	
	Ti	PEEK	Ti	PEEK	Ti	PEEK	Ti	PEEK
**N = 1**	0.0077	0.0106	0.0093	0.0075	0.0087	0.0069	0.0124	0.0065
**N = 2**	0.0080	0.0079	0.0061	0.0064	0.0076	0.0084	0.0122	0.0106
**N = 3**	0.0072	0.0074	0.0069	0.0107	0.0108	0.0044	0.0063	0.0075
**N = 4**	0.0066	0.0068	0.0057	0.0086	0.0050	0.0107	0.0071	0.0129
**N = 5**	0.0083	0.0075	0.0104	0.0052	0.0060	0.0051	0.0087	0.0121
**N = 6**	0.0072	0.0063	0.0096	0.0094	0.0077	0.0138	0.0062	0.0089
**N = 7**	0.0069	0.0057	0.0054	0.0074	0.0081	0.0083	0.0079	0.0056
**Mean**	0.0074	0.0074	0.0075	0.0079	0.0077	0.0082	0.0087	0.0092

**Table 2:** The statistical analysis of the collected data for the experiments for the abutment, day, and abutment * day.

Tests of Between-Subjects Effects						
Dependent Variable: OD			Optical Density/Surface Area (1/mm^2^)			
Source	Type III Sum of Squares	df		Mean Square	F	Sig.
**Corrected Model**	1.960 × 10^−5 a^	7		2.800 × 10^−6^	0.574	0.774
**Intercept**	0.004	1		0.004	746.317	<0.0001
**Abutment**	1.509 × 10^−6^	1		1.509 × 10^−6^	0.309	0.581
**Day**	1.755 × 10^−5^	3		5.851 × 10^−6^	1.199	0.320
Abutment * Day	5.399 × 10^−7^	3		1.800 × 10^−6^	0.037	0.990
**Error**	0.000	48		4.881 × 10^−6^		
**Total**	0.004	56				
**Corrected total**	0.000	55				

a. R Squared = 0.077 (Adjusted R Squared = −0.057). Analysis was conducted using Two-Way ANOVA and post hoc analysis. The significance difference at α = 0.05.

## Data Availability

The study data are available on request from the corresponding author.
